# *Dirofilaria immitis* Causing Flexor Tenosynovitis of the Hand: A Rare Case Report

**DOI:** 10.1155/cro/3569396

**Published:** 2026-07-22

**Authors:** Jamie C. Henzes, John T. Rich

**Affiliations:** ^1^ Department of Orthopaedic Surgery, UPMC Central PA, Harrisburg, Pennsylvania, USA; ^2^ Lehigh Valley Health Network, Dickson City, Pennsylvania, USA, lvhn.org

**Keywords:** *Dirofilaria*, flexor tenosynovitis, heartworm, pathology

## Abstract

This case describes a previously underreported case of flexor tenosynovitis. A 23‐year‐old male slaughterhouse employee presented with several weeks of increased pain and swelling in their left third digit. They failed nonoperative management, including anti‐inflammatory medication and oral steroids, followed by corticosteroid injection. Intraoperative pathology demonstrated subacute flexor tenosynovitis due to a rare parasitic infection of *Dirofilaria immitis*. *Dirofilaria immitis*, a parasitic roundworm commonly found in dogs, rarely causes zoonotic infections in humans. In cases when this parasite infects humans, it primarily impacts pulmonary, ocular, and subcutaneous tissues. This diagnosis was ultimately only able to be made due to pathologic analysis, as they had negative cultures. This case highlights that tissue pathology is essential in atypical infections, especially culture‐negative tenosynovitis.

## 1. Introduction

Flexor tenosynovitis is an enclosed infection of the synovial sheath surrounding the flexor tendons of the digits. This comprises 2.5%–9% of all hand infections. Typical flexor tenosynovitis is caused by skin flora, such as *Staphylococcus* and *Streptococcus* species, after direct trauma. However, atypical organisms, including gram‐negative rods, fungi, and parasites, can cause chronic presentations, especially in immunocompromised individuals [1]. Complications of flexor tenosynovitis include scarring, digit contraction, and, in severe cases, necrosis requiring amputation [2]. Our case presents an atypical presentation for a young male slaughterhouse employee who was ultimately diagnosed and treated with a subacute flexor tenosynovitis where pathology revealed the cause to be the parasite *Dirofilaria immitis*. To our knowledge, this is the first case reported of flexor tenosynovitis in a hand resulting from a *Dirofilaria immitis* infection.

The *Dirofilaria immitis* parasite transfers disease from animals to humans through mosquitoes as the vector, causing zoonotic disease. This parasite has been shown to infect subcutaneous tissues within human hosts. *Dirofilaria immitis* is a specific type of roundworm most known for causing heartworm in dogs, having a 6.4% incidence rate in the United States and 10.9% globally [3]. Humans are dead‐end hosts for these parasites, making infections rare.

This case presents the unique treatment course that the patient underwent in this rare case. We highlight the clinical relevance of having a high index of infection in atypical presentations and obtaining pathologic specimens. This infection can be misdiagnosed as chronic tendinitis, leading to unnecessary steroid injections, possible delayed surgery, and permanent functional deficits. Successful treatment was only able to be accomplished due to having a broad differential diagnosis, close monitoring of the patient′s conditions, and obtaining pathologic specimens in addition to operative cultures. Written informed consent was obtained by the patient for publication of this case report and accompanying images.

## 2. Case Presentation

A 23‐year‐old, right‐hand‐dominant male with an unremarkable past medical history presented to the outpatient orthopedic office with 2 months of reported left‐hand pain. The patient had recently begun a new job working in a slaughterhouse, involving heavy repetitive lifting during the 6 months prior to his onset of symptoms. They denied any injury or inciting event. The patient had initially been seen in employee health, at which time they were diagnosed with tendinitis and started on anti‐inflammatory medications with minor improvement.

**Figure 1:**
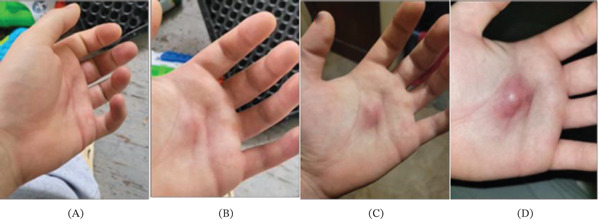
Serial clinical images of the palmar surface of the left hand demonstrating the progression of symptoms. (A) Initial presentation showing minimal erythema. (B) Two‐week follow‐up demonstrating increasing erythema and edema of the palm. (C) Four weeks after initial presentation showing worsening erythema and development of nodularity. (D) Eight weeks after initial presentation demonstrating marked progression of erythema and edema, prompting operative intervention.

**Figure 2:**
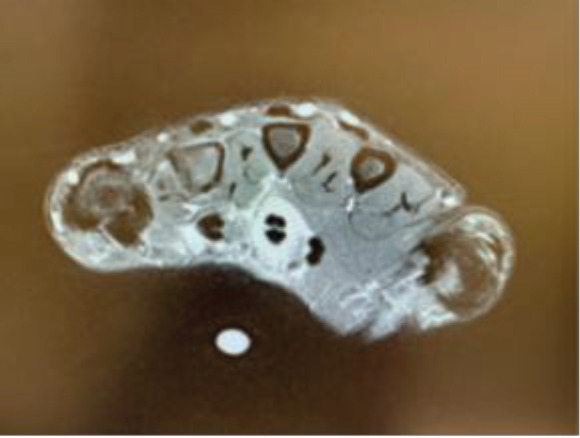
T1‐weighted axial MRI of the left hand demonstrating marked peritendinous edema surrounding the flexor tendon sheaths, most pronounced in the third digit.

After 2 weeks of unimprovement on the anti‐inflammatories, the patient presented to our orthopedic clinic for persistent hand pain and swelling (Table 1). On physical examination, swelling of the left third digit was noted. The left third digit was tender to palpation over the A1 pulley; however, there was minimal pain with active or passive range of motion. This clinical exam feature is commonly seen in tendinitis and is unusual for tenosynovitis. A chronic flexion contracture of less than 10° was additionally noted at the proximal interphalangeal (PIP) joint (Figure 1A,B). The patient denied any recent illness and had no signs or symptoms of a local or systemic infection other than swelling of the digit. A magnetic resonance imaging (MRI), which had been previously ordered by their primary care physician, demonstrated peritendinous edema without tear or abscess (Figure 2).

**Table 1:** Timeline of patient clinical course, including time points, symptoms, differential diagnosis, and treatment at each individual time point, from initial presentation to resolution of symptoms.

Time point	Symptoms	Differential diagnosis	Intervention
Employee health visit	Two months of left finger pain, swelling, tender to palpation	Tendinitis, chronic tenosynovitis	Two‐week course NSAIDs
Two weeks (initial orthopedic visit)	Persistent swelling, tenderness, pain after NSAIDs	Tendinitis, chronic tenosynovitis	Oral steroids (methylprednisolone)
Four weeks (2‐week orthopedic follow‐up)	Persistent swelling, tenderness, pain after oral steroids	Tendinitis, chronic tenosynovitis	Steroid injection (1% lidocaine and 1 cc triamcinolone)
Six weeks (2‐week orthopedic follow‐up)	Persistent swelling, tenderness, pain after steroid injection	Chronic tenosynovitis	Operative irrigation and debridement (tissue cultures/pathology obtained)
Six weeks and 2 days (2 days postoperative)	Significant improvement in symptoms	Pathology reveals *Dirofilaria*	Referred to infectious disease (gives ivermectin and 1‐month NSAIDs)
Two years	Complete resolution of symptoms without recurrence	—	—

Based upon imaging and clinical examination, it was determined that flexor tendinitis was the most likely diagnosis. Treatment options were discussed including oral steroids or possible injections, both to reduce what was believed to be inflammation of the flexor sheath. It was decided to start treatment with oral steroids, methylprednisolone, prior to attempting a steroid injection. At his 2‐week follow‐up appointment, the patient reported minimal relief and continued swelling, with unchanged range of motion (Figure 1C). At that visit, the patient elected to undergo a corticosteroid injection into the third digit flexor tendon sheath in standard fashion with 1 cc of 1% lidocaine and 1 cc triamcinolone (Table 1).

They returned 4 weeks later with increased erythema, edema, and now painful active and passive range of motion of the left third digit (Figure 1D). At this stage, a subacute atypical flexor tenosynovitis infection was suspected, with *Mycobacterium* of primary concern, given the prolonged subacute presentation. The following day, the patient was taken to the operating room for surgical irrigation and debridement (I&D). The hand was marked, prepped, and draped in standard fashion. A Bruner incision was made along the volar aspect of the third digit, which revealed significant synovitis and purulent fluid within the flexor tendon. Synovectomy of the third digit flexor tendon was performed, including removal of tissue proximal to the A1 pulley. Tissue samples were sent for cultures (aerobic, anaerobic, fungal, and acid‐fast bacilli [AFB]) and pathology. After thorough I&D, the wound was closed with nylon sutures, and dry sterile dressings were applied. The patient tolerated the procedure without issue, was advised to avoid heavy use of their left hand, and was discharged home the same day.

**Figure 3:**
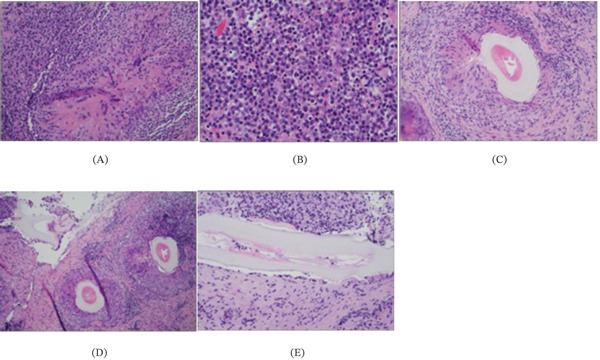
Microscopic examination demonstrating marked inflammation with granuloma formation. (A) Histologic section demonstrating an inflammatory infiltrate predominantly composed of lymphocytes and plasma cells. (B) Higher magnification demonstrating lymphocytes and plasma cells with scattered eosinophils and neutrophils. (C) Transverse section of the parasite demonstrating visualization of the lumen. (D) Transverse section demonstrating two distinct lumens within the parasite. (E) Longitudinal section of the parasite, with histologic features consistent with *Dirofilaria immitis*.

Evaluation of the patient 2 days postoperatively demonstrated improvement in the erythema and swelling. Interestingly, the gram stain identified rare gram‐positive cocci, but there was no growth of the cultures. Surprisingly, the tissue pathology was morphologically consistent with *Dirofilaria immitis* or related species (Figure 3). The patient was referred to an infectious disease specialist, who treated the patient with a one‐time dose of 250 mcg/kg ivermectin, as well as a 1‐month course of nonsteroidal anti‐inflammatory medication. The third digit incision healed well without complication and had minimal stiffness. The patient was followed intermittently for over 2 years and has had no issues or limitations with his digit (Table 1).

## 3. Discussion

Human *Dirofilaria immitis* is a zoonotic infection caused by roundworms of the genus *Dirofilaria*. This parasite is transmitted by mosquitoes and commonly uses both domesticated and wild animals as reservoirs [4]. The life cycle starts with the adult *Dirofilaria*, which produces immature parasites that circulate in the peripheral blood until the host′s blood is consumed by a mosquito. The mosquito will then transfer the parasite to a new host, thus allowing it to mature and repeat the cycle [5]. Human infection occurs when a mosquito penetrates the skin and transmits *Dirofilaria* microfilariae into the bloodstream. Transmission in human hosts ends, however, as the larvae die before reaching sexual maturity. Therefore, humans are dead‐end hosts to *Dirofilaria*. However, shared environments between humans and animals, specifically domesticated dogs, may account for increased opportunities of dog‐to‐human infection [6].

Adult *Dirofilaria* have been shown to migrate within human subcutaneous tissues for months before they die, leading to vasculitis and granulomatous reactions [6]. Pulmonary nodules have been the most frequent location of symptomatic *Dirofilaria* in humans. However, Theis showed that extrapulmonary tissue involvement can include the head, neck, torso, arms, legs, and even the genitals [7]. No published reports of *Dirofilaria* within digital flexor compartments were discovered during our most recent literature review. However, Ramirez et al. described a case of *Dirofilaria* infection located within the forearm that produced a median nerve neuropathy [6]. Similar to our case, there was an initial 2‐month delay in operative treatment due to suspicion of flexor tenosynovitis. Subsequently, they were diagnosed with a giant cell tumor, leading to surgical excision, of which the pathologic tissue demonstrated atypical parasitic infection. Diagnosis of *Dirofilaria* is made using histopathologic analysis with species identification based on microscopic features [8]. Ivermectin therapy has additionally been shown to treat conjunctival and subcutaneous infection [9].

Flexor tenosynovitis can present as acute, subacute, or chronic infection. Acute infection is considered an orthopedic emergency, as prolonged infection can lead to necrosis and the need for amputation of the infected digit. It commonly presents with the following cutaneous trauma that leads to rapid progression of infection. Clinical examination may show the four signs referenced by Dr. Kanavel: uniform digit swelling, flexed digit posturing, tenderness to palpation over the affected tendon sheath, and marked pain with passive digit extension [10]. Subacute and chronic infections, as in this case, usually do not present initially with the classic Kanavel signs.

Acute bacterial skin flora, commonly *Staphylococcus aureus* and *Streptococcus*, is discovered on culture and initially treated empirically with broad‐spectrum antibiotics [1]. If no improvement is noted within 24 h, operative I&D of the flexor tendon sheath is completed, followed by culture‐specific IV antibiotics. For subacute and chronic infections, initial conservative treatment can include steroids and antibiotics. Surgical debridement is necessary in subacute and chronic cases that fail to respond to initial conservative measures. Ivermectin was given under the direction of infectious disease as it is an antiparasitic that selectively binds to glutamate‐gated chloride channels, causing hyperpolarization and paralysis [7].

This case highlights an exceptionally rare presentation of subacute flexor tenosynovitis caused by *Dirofilaria immitis*, a zoonotic parasitic infection not previously reported within the flexor tendon sheath. The patient′s atypical presentation, including minimal pain with motion, chronic swelling, and lack of classic Kanavel signs, initially mimicked flexor tendinitis and led to treatment with anti‐inflammatory medications and corticosteroids. However, progression despite conservative management ultimately prompted surgical intervention, which revealed significant synovitis and purulence. Importantly, routine operative culture remained negative, and the diagnosis was only established through histopathologic evaluation of the operative tissue specimens. This case emphasizes the importance of maintaining a broad differential diagnosis in persistent or atypical hand infections, especially in patients with unique occupational or environmental exposures. It also demonstrates the critical diagnostic value of obtaining tissue pathology in addition to standard cultures during operative treatment.

## 4. Conclusion

This case represents the first reported instance of flexor tenosynovitis associated with *Dirofilaria immitis* infection within the digital flexor tendon sheath. The patient′s atypical, indolent presentation and absence of Kanavel signs initially mimicked flexor tendinitis. This resulted in treatment with corticosteroids before the underlying diagnosis was recognized. Clinically, this case underscores the importance of maintaining a broad differential diagnosis in patients with persistent or progressive tendinitis who fail to respond to conventional treatments. Furthermore, it highlights the limitations of culture‐based testing alone, as diagnosis was achieved through histopathologic identification. Early surgical intervention combined with routine submission of tissue pathology may prevent delays in diagnosis, avoid ineffective treatments, and improve functional outcomes in patients with atypical or culture‐negative flexor tenosynovitis.

## Author Contributions

**Jamie C. Henzes:** investigation, conceptualization, visualization, writing – original draft, writing – review and editing. **John T. Rich:** supervision, resources, writing – review and editing.

## Funding

No funding was received for this manuscript.

## Ethics Statement

Institutional Review Board (IRB) approval was obtained prior to publication of this case report.

## Consent

Written informed consent was obtained by the patient for the publication of this case report and accompanying images. All identifying information has been removed to protect patient privacy.

## Conflicts of Interest

The authors declare no conflicts of interest.

## Data Availability

No new data were created or analyzed during this study. Data sharing is not applicable to this article.
